# Novel Dicarboxylate Selectivity in an Insect Glutamate Transporter Homolog

**DOI:** 10.1371/journal.pone.0070947

**Published:** 2013-08-07

**Authors:** Hui Wang, Avi M. Rascoe, David C. Holley, Eric Gouaux, Michael P. Kavanaugh

**Affiliations:** 1 Vollum Institute, Oregon Health & Science University, Portland, Oregon, United States of America; 2 Center for Structural and Functional Neuroscience, University of Montana, Missoula, Montana, United States of America; 3 Howard Hughes Medical Institute, Oregon Health & Science University, Portland, Oregon, United States of America; University of Texas Health Science Center, United States of America

## Abstract

Mammals express seven transporters from the SLC1 (solute carrier 1) gene family, including five acidic amino acid transporters (EAAT1–5) and two neutral amino acid transporters (ASCT1–2). In contrast, insects of the order Diptera possess only two SLC1 genes. In this work we show that in the mosquito Culex quinquefasciatus, a carrier of West Nile virus, one of its two SLC1 EAAT-like genes encodes a transporter that displays an unusual selectivity for dicarboxylic acids over acidic amino acids. In eukaryotes, dicarboxylic acid uptake has been previously thought to be mediated exclusively by transporters outside the SLC1 family. The dicarboxylate selectivity was found to be associated with two residues in transmembrane domain 8, near the presumed substrate binding site. These residues appear to be conserved in all eukaryotic SLC1 transporters (Asp444 and Thr448, human EAAT3 numbering) with the exception of this novel C. quinquefasciatus transporter and an ortholog from the yellow fever mosquito Aedes aegypti, in which they are changed to Asn and Ala. In the prokaryotic EAAT-like SLC1 transporter DctA, a dicarboxylate transporter which was lost in the lineage leading to eukaryotes, the corresponding TMD8 residues are Ser and Ala. Functional analysis of engineered mutant mosquito and human transporters expressed in Xenopus laevis oocytes provide support for a model defining interactions of charged and polar transporter residues in TMD8 with α-amino acids and ions. Together with the phylogenetic evidence, the functional data suggest that a novel route of dicarboxylic acid uptake evolved in these mosquitos by mutations in an ancestral glutamate transporter gene.

## Introduction

In mammals, SLC1 (solute carrier family 1) transporter genes can be divided into two broad functional subclasses made up of five acidic (or excitatory) amino acid transporters (EAAT1–5) and two neutral amino acid transporters (ASCT1–2)[1]–[4]. Structures of the archael EAAT transporter Glt_ph_ have been resolved in several different conformational states [5]–[8], and in conjunction with functional studies on homologous mammalian EAATs, the data have provided insights into structural mechanisms in these transporters [9]. One functionally critical structural feature is transmembrane region 8 (TMD8), which forms part of the substrate binding domain [5]. This domain contains several conserved charged residues, including D440, D444, R447, and D455 (human EAAT3 numbering). These residues appear to play critical roles because individual mutations of any of them lead to severe disruptions in glutamate transport [10]–[13]. While these polar and charged TMD8 residues tend to be highly conserved in the SLC1 family, extensive gene duplication may have reduced selective pressure against mutation, and allowed an expanded functional repertoire of these transporters to evolve. For example, neutralizing mutations of the R447 residue in TMD8 change the transporter’s selectivity from acidic to neutral amino acids, and in the mammalian SLC1 neutral amino acid transporter subfamilies ASCT1 and ASCT2, a Ser or Cys residue is found in place of Arg at this position [12], [14].

There is wide variation in the number of SLC1 genes across species [2]. In contrast to mammals, which express a total of seven SLC1 genes, insects of the order Diptera express only two. In Drosophila, one of these (dEAAT1) mediates uptake of the excitatory amino acids aspartate and glutamate [15], while the other (dEAAT2) mediates preferential uptake of aspartate and taurine over glutamate [16]. Blood-feeding mosquito species including Anopheles gambiae, Aedes aegypti and Culex quinquefasciatus, vectors for disease-causing parasites and viruses, also follow the Dipteran pattern of harboring two SLC1 genes. A transporter from Aedes aegypti called AeaEAAT, first cloned and expressed by Umesh et al. [17], displays EAAT-like properties and mediates uptake of L-aspartate and L-glutamate with similar K_m_ values. This Aedes transporter shares 48% identity with dEAAT1 and 39% with dEAAT2. Culex quinquefasciatus, a carrier of the West Nile virus, expresses an ortholog of the Aedes aspartate/glutamate transporter that is 88% identical (gene accession number XP_001842238). In this work, we expressed and characterized the other SLC1 transporter from Culex (accession number XP_001842239). This transporter (hereafter called CuqDCT) displayed a dramatically altered substrate selectivity, preferring dicarboxylic acids over the acidic α-amino acids glutamate and aspartate. We provide evidence that this selectivity change is associated with coding changes in the mosquito genes corresponding to conserved residues in the middle of TMD8 (D444 and T448 in human EAAT3). These residues are separated by approximately one helix turn and are strictly conserved in all eukaryotic SLC1 members aside from the Culex dicarboxylate transporter and its ortholog in Aedes aegypti. The prokaryotic SLC1 transporter DctA is a dicarboxylate transporter not present in eukaryotes [18]–[21]. Like the Culex and Aedes transporters, the corresponding TMD8 residues in DctA differ from the conserved eukaryotic residues. The functional and phylogenetic data from this study provide insights into both the mechanisms underlying amino acid and ion selectivity in this transporter family as well as into the evolutionary biology of nutrient transport in some mosquito species.

## Materials and Methods

### Ethics Statement

Xenopus laevis used in this study were treated in a manner to minimize suffering, and were anesthetized with tricane prior to oocyte removal in accordance with NIH and University of Montana IACUC regulations (IACUC protocol approval 065-11MKBMED-122111).

### Molecular Biology

The mosquito gene encoding CuqDCT (accession number XP_001842239) was synthesized and sub-cloned in-frame with C-terminal GFP [22] into plasmid pCDNA3.1 (kindly provided by K. Duerr and T. Friedrich). RNAs encoding wild-type and mutant CuqDCT and EAAT3 (in pGEM) were transcribed using the mMessage mMachine T7 Ultra kit (Ambion). Defolliculated stage V–VI Xenopus oocytes were kindly provided by D.C. Dawson and C. Alexander.

### Electrophysiology

Oocytes were injected with 50 ng of cRNAs, and voltage-clamp current recordings were made 3–5 days later. Recording electrodes (0.2–1.0 MΩ) were filled with 3 M KCl. Recording solution (frog Ringer) contained: 96 mM NaCl, 2 mM KCl, 1 mM MgCl_2_, 1.8 mM CaCl_2_, 5 mM HEPES pH 7.5. L-Glu, L-Asp, succinate, α-ketoglutarate and malate (Sigma) 100–1000×concentrated stock solutions were prepared in the recording solution above. Oocytes were voltage-clamped at −60 mV unless indicated, and data were recorded with Molecular Devices amplifiers and analog-digital converters interfaced to a PC. Data was acquired and analyzed with Axograph and Kaleidagraph software.

### Uptake Assays

Oocyte radiolabel transport assay were carried out 72 hrs post-injection. Oocytes expressing wild-type or mutant transporters were incubated with indicated concentrations of [^3^H]-labeled L-glutamate (0.5 Ci mmol^−1^) or succinate (0.6 Ci mmol^−1^) in Ringer. Uptake was stopped by washing 3× with 4°C buffer, then oocytes were lysed in 0.1% Triton X-100, and radioactivity was measured by liquid scintillation spectroscopy.

### Computational Modeling and Sequence Alignment

Structural models for both CuqDCT and human EAAT3 were generated using the SwissProt server [23] based on the structure of Glt_ph_ in an outward-facing, substrate-occluded conformation (PDB code 2NWL). All structure figures were generated with PyMOL (DeLano Scientific). The schematic diagrams of protein-ligand interactions were generated by LIGPLOT [24]. Multiple sequence alignments were performed by using ClustalX2 [25] and edited in Jalview [26]. The phylogenetic tree was drawn by NJplot [27] based on alignment from ClustalX2.

## Results

### SLC1 Genes in Mosquito Species

**Figure 1:**
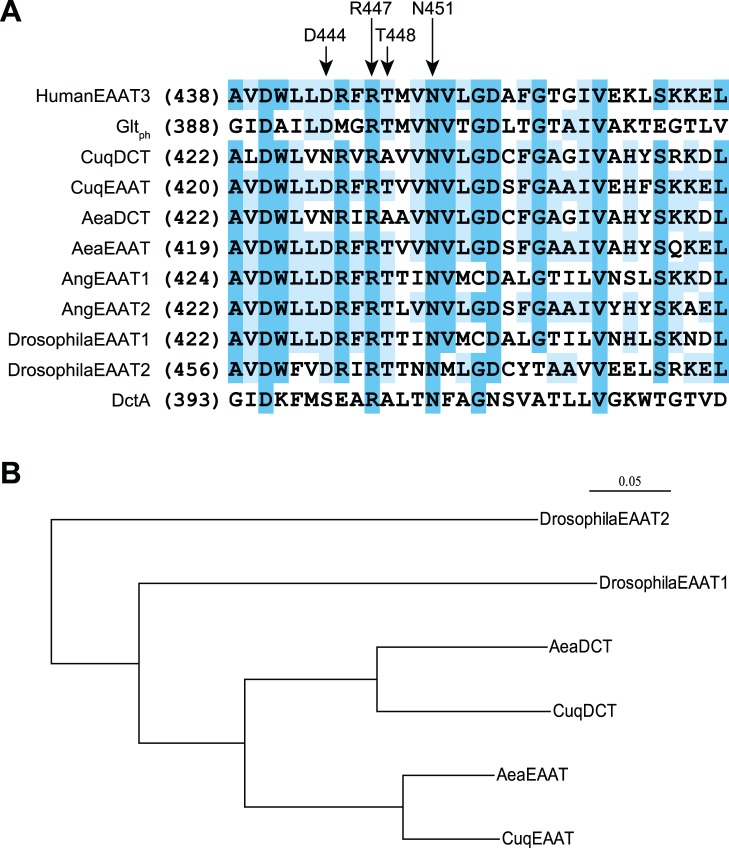
Sequence analysis of eukaryotic and prokaryotic SLC1 transporters. (A) Sequence alignment of TMD8 region from human, insect, bacterial, and archael transporters. CuqDCT, CuqEAAT, AeaDCT, and AeaEAAT represent the EAAT and DCT orthologs from Culex quinquefasciatus and Aedes aegypti, respectively. AngEAAT1 and AngEAAT2 represent the transporters from Anopheles gambiae. Drosophila EAAT1/2 are from Drosophila melanogaster, Glt_ph_ from Pyrococcus horikoshii, and DctA is from Bacillus subtilis. Highlighted TMD8 residues involved in substrate binding are labeled above using human EAAT3 numbering. R447 and N451 are conserved in all EAAT-like orthologs, while D444 and T448 are changed to asparagine and alanine, respectively in the CuqDCT and AeaDCT mosquito transporters. Sequence alignments were performed with ClustalX2 and Jalview. (B) The phylogenetic relationship of the EAAT and DCT proteins from Culex quinquefasciatus and Aedes aegypti mosquito species together with the two EAATs from Drosophila (ClustalX2 based alignment).

Genbank sequence analysis using BLAST indicates that mosquito species possess only two SLC1 genes, like other members of the order Diptera. One of these genes, from the mosquito Aedes aegypti, has been previously expressed and shown to encode a transporter (AeaEAAT) with typical EAAT-like activity [17]. One of the predicted SLC1 gene products from the related species Culex quinquefasciatus (accession code XP_001842238) shares 88% sequence identity with AeaEAAT (accession code XP_001649287), and we termed this apparent ortholog CuqEAAT. The other SLC1 genes in Culex (XP_001842239) and Aedes (XP_001656354) also appear to be orthologs with predicted gene products sharing 78% identity. We noted that these latter predicted transporters contained an Asn residue at a position in TMD8 (N428) that is otherwise stringently conserved as Asp in the SLC1 gene family (D444, human EAAT3 numbering; Fig. 1A, Fig. S1). The structure of the homologous archael transporter Glt_Ph_ indicates that this conserved Asp residue lies near the middle of TMD8 near the substrate binding pocket [5], [6] and its mutation is known to disrupt EAAT activity [10], [11]. Notably, a similar neutralizing change (Asp to Ser) is seen in the corresponding residue of the prokaryotic SLC1 subfamily of dicarboxylate transporters known as DctA (Fig. 1A). These structural differences and the functional data described below led us to designate the second pair of orthologous transporters from A. aegypti and C. quinquefasciatus as AeaDCT and CuqDCT respectively. Alignment of TMD8 sequences (Fig. 1A) show another change in the DCT orthologs at a conserved Thr residue approximately one helix turn below (T448, hEAAT3 numbering). Other conserved EAAT residues in TMD8 are also present in C. quinquefasciatus and A. aegypti, including D440, N451, R447, and D455 (hEAAT3 numbering). The homologies across species between AeaDCT and CuqDCT and AeaEAAT and CuqEAAT were higher than the homologies between the SLC1 genes within each mosquito species (78–88% vs 63–66% predicted protein identity, respectively; Table S1). We did not detect equivalent TMD8 changes in other mosquito SLC1 genes, suggesting that the C. quinquefasciatus and A. aegypti mutations occurred prior to the divergence of these two species from each other, but after the divergence of their progenitor from other mosquitos (Fig. 1B).

### Transport Properties of CuqDCT

**Figure 2:**
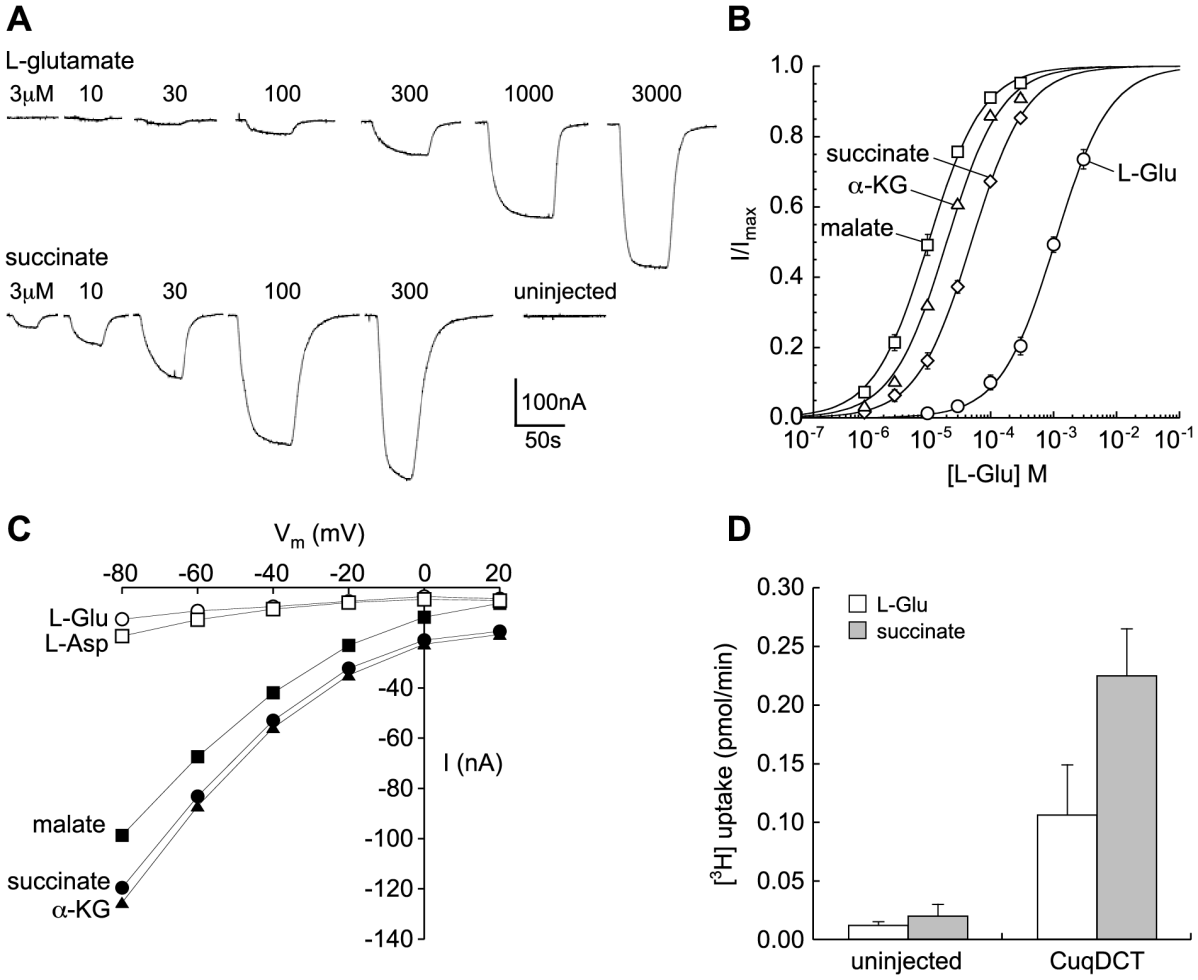
Transport activity of wild type CuqDCT. (A) Current recordings from a representative voltage-clamped oocyte (−60 mV) expressing wild-type CuqDCT showing concentration-dependence of responses induced by superfusion of L-glutamate (top) or succinate (bottom) at concentrations indicated above trace. Lack of response to 300 µM succinate in representative uninjected oocyte is also shown. (B) Summary of kinetic data for oocytes expressing for wild type CuqDCT showing fits of data to the Michaelis-Menten equation reflecting Km values for dicarboxylic acids approximately two orders of magnitude lower than for L-Glu. No significant difference were observed in I_max_ values. (C) CuqDCT-mediated currents clamped at different voltages were induced by the 100 uM L-glutamate, L-aspartate, succinate, malate and α-ketoglutarate. (D) Radiolabel [^3^H] succinate and L-glutamate (10 uM) uptake assay for oocytes expressing wild type CuqDCT. Uninjected oocytes were used as control. Error bars represent SEM, n≥3.

**Figure 3:**
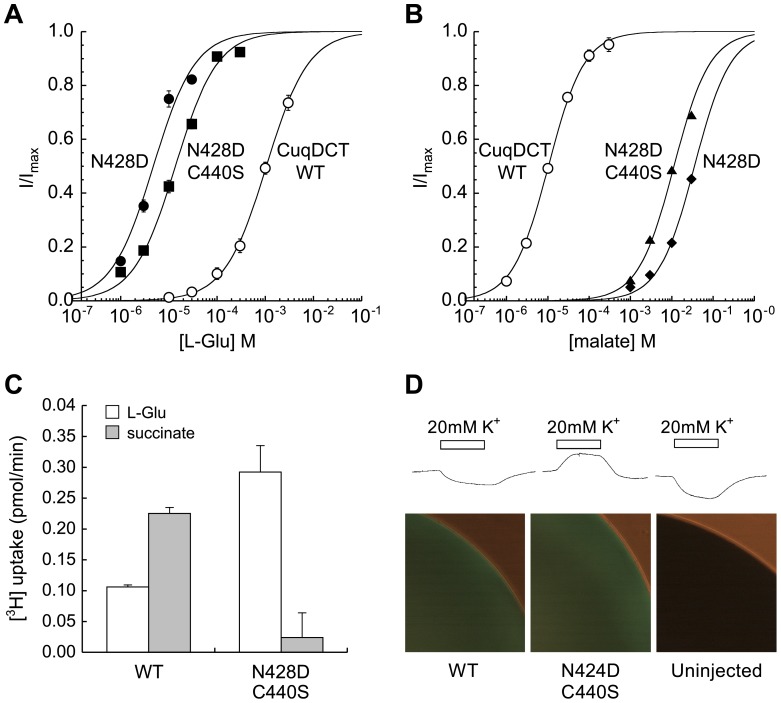
The mutation N428D in CuqDCT either alone or in the C440S background changed substrate selectivity. The apparent affinities of the mutants for L-glutamate (A) were increased approximately two orders of magnitude (K_m_ shift from 1.1±0.1 mM to 13.8±1.3 µM), while the apparent affinity for malate (B) was decreased (K_m_ shift from 11±1 µM to 8.8±1.5 mM). (C) Comparison of the uptake of 10 µM [^3^H] L-glutamate and [^3^H] succinate for oocytes expressing the wild type CuqDCT or double mutant N428D/C440S. (D) Inward currents are induced by addition of 20 mM extracellular K^+^ in uninjected oocytes and in oocytes expressing wild-type CuqDCT, while oocytes expressing comparable levels of N428D/C440S mutant (fluorescence micrographs below) respond with outward currents (n≥3 for each group).

The CuqDCT gene was synthesized and fused in-frame with C-terminal EGFP. Following injection of cRNA, transporter expression was screened in oocytes by epifluorescence microscopy and by two-microelectrode voltage clamp recording (Fig. 2, 3). At −60 mV, the concentration threshold for detecting L-Glu responses was 10 µM, while application of equimolar dicarboxylates induced significantly larger currents that were not seen in uninjected control oocytes (Fig. 2A). The currents induced by all substrates were concentration-dependent and saturable (Fig. 2A,B). The transporter exhibited a significantly higher apparent affinity for succinate than for L-glutamate (K_m_ values of 50.8±2.3 µM and 1085±96 µM, respectively). Other dicarboxylic acids tested induced currents similar to succinate with high apparent affinity, including malate (10.5±0.5 µM) and α-ketoglutarate (20.2±1.2 µM). Citric acid did not induce detectable currents at concentrations up to 1 mM. The maximum current amplitudes induced by saturating concentrations of dicarboxylic acids and L-glutamate were not significantly different in the same oocytes (paired-T test). The voltage-dependence of currents induced by dicarboxylic acids and acidic amino acids each displayed inwardly rectifying behavior (Fig. 2C). Uptake of 10 µM [^3^H] L-glutamate and [^3^H] succinate confirmed that succinate was transported preferentially in oocytes expressing CuqDCT (Fig. 2D).

### Structural Basis of Altered Substrate and Ion Selectivity in CuqDCT

Based on the phylogenetic analysis above, we made the N428D mutation in CuqDCT (D444 EAAT3 numbering) to test the role of this residue in substrate selectivity. We also made mutations in two other Culex TMD8 residues (A432T and C440S) that varied from conserved EAAT patterns (Fig. 1A). The mutation N428D on its own had a large effect, increasing the apparent affinity of CuqDCT for L-glutamate by approximately two orders of magnitude (K_m_ shift from 1.1±0.1 mM to 13.8±1.3 µM; Fig. 3A), and a similarly large decrease in the apparent affinity of the transporter for dicarboxylates (K_m_ shift for malate from 11±1 µM to 8.8±1.5 mM; Fig. 3B). The double mutant CuqDCT N428D/C440S exhibited properties similar to N428D, but expressed at higher levels (the average maximum currents induced by glutamate in the single and double mutants was approximately 7% and 20% of the maximum dicarboxylate currents in the wild-type, respectively). Comparison of glutamate and succinate radiolabel transport rates confirmed that a change in selectivity of substrate transport was consistent with that inferred from electrophysiological measurements (Fig. 3A–D). These results are consistent with the possibility that phenotypic conversion of an ancestral mosquito glutamate transporter (with consensus EAAT residues D428 and T432) to a dicarboxylate transporter could have occurred by sequential mutation of T432A (maintaining high affinity glutamate transport; Fig. 3A) followed by the D428N mutation resulting in the CuqDCT dicarboxylate transporter phenotype.

Eukaryotic EAATs utilize potassium gradients by countertransporting K^+^ during an L-Glu transport cycle [28]. Addition of extracellular potassium induces voltage-dependent outward currents associated with reverse transport in EAAT3 [29]. In addition to differences in substrate selectivity, prokaryotic DctA transporters differ from EAATs by virtue of the fact that they do not utilize K^+^ countertransport for substrate uptake [21]. Superfusion of 20 mM K^+^ on voltage-clamped oocytes expressing wild-type CuqDCT induced small inward currents not different from uninjected oocytes, whereas EAAT-like outward currents were seen in oocytes expressing similar levels of the N428D/C440S mutant transporters (Fig. 3D).

### Corresponding TMD8 Residues in EAAT3 are Associated with Glutamate/Dicarboxylate Selectivity

**Figure 4:**
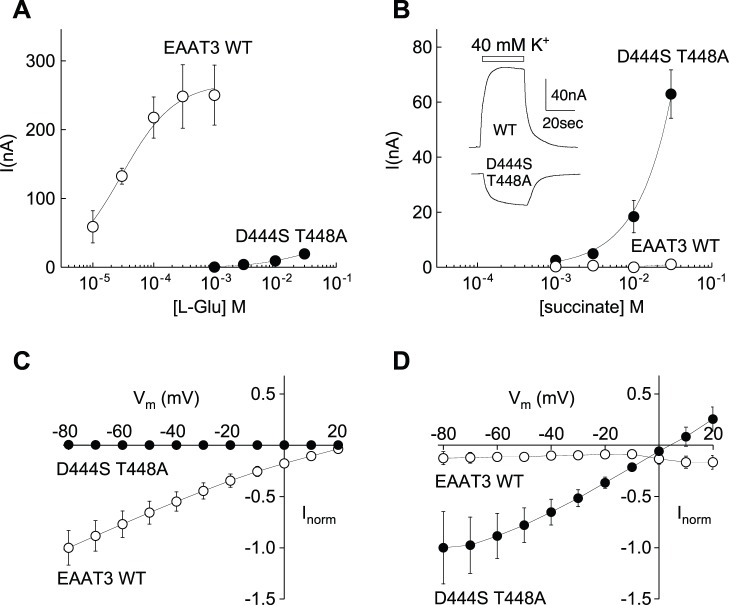
Comparison of EAAT3 wild-type and D444S/T448A mutant transporter currents. Concentration-dependence of currents in response to L-glutamate (A) or succinate (B) in oocytes expressing wild-type EAAT3 or mutant D444S/T448A. Curves show fit of data to the Michaelis-Menten equation. No outward current was induced by addition of extracellular K^+^ in oocytes at −20 mV expressing mutant D444S/T448A, in contrast to wild type EAAT3 (B inset). Comparison of the voltage-dependence of wild type EAAT3 and D444S/T448A mutant currents induced by either 1 mM L-glutamate (C) or 10 mM succinate (D). In C and D, current amplitudes were normalized to the response at −80 mV. Error bars represent SEM, n≥3.

In order to gain further insight into the role of TMD8 residues in substrate selectivity, we made mutations in the corresponding residues D444 and T448 in the human neuronal glutamate transporter EAAT3. In accord with work from Teichman and Kanner, we found that EAAT3 transporters with the mutations D444S or D444N did not mediate steady-state transport currents in response to superfusion of 1 mM L-Glu or L-Asp, nor to superfusion of 10 mM succinate, malate, or α-ketoglutarate, although the dicarboxylates were previously shown to block Na^+^-dependent transient currents [10]. The single mutation T448A in EAAT3 reduced glutamate transport to levels to approximately 20% of wild-type, but did not change the transporter’s selectivity [10]. In contrast, the mutation T448A in conjunction with D444S did confer the ability to mediate dicarboxylic acid-induced steady-state transport currents and dramatically reduced the transporter’s apparent affinity for L-Glu/Asp (Fig. 4). The double mutant correspondingly mediated selective uptake of [^3^H]succinate, which was not observed for wild-type EAAT3 (see supporting figure S2). The voltage-dependence of currents induced by dicarboxylates in the EAAT3 D444S/T448A mutant showed that the current reversal potential was shifted approximately 15–20 mV negative relative to EAAT3, suggesting that the ratio of the uncoupled chloride current [30] to the coupled transport current may be increased in the mutant. Reducing the extracellular Cl^-^ concentration to 60 mM by substitution with the impermeant anion gluconate shifted the reversal potential of the succinate-induced current 18±2 mV (n = 5), confirming a Cl^−^ conductance associated with dicarboxylate transport in the mutant. In contrast to Cl^−^, glutamate transport is thermodynamically coupled to K^+^ countertransport, and in oocytes expressing either wild-type EAAT3 [29] or the single mutant T448A elevation of K^+^ in the perfusate induced outward currents. In oocytes expressing the double mutant D444S/T448A, elevated K^+^ induced inward currents similar to uninjected oocytes rather than outward currents as seen in oocytes expressing wild-type EAAT3 (Fig. 4B). This effect is the same as with the corresponding mutation N424D in the mosquito transporter, which conferred an outward current in response to raised extracellular K^+^ (Fig. 3D) and highlights a possible role of this aspartate residue in mediating K^+^ countertransport.

## Discussion

As noted by Broer [31], alterations in amino acid transporter selectivity within gene families are common and often appear to result from discrete mutations in concert with changing physiological demands during evolution. The genetic divergence of mosquitos approximately 250 million years ago has proceeded significantly faster than the divergence of other Dipteran genomes, perhaps in part because of differences in selective pressures and metabolic adaptations associated with blood feeding [32]. The Drosophila SLC1 family members share approximately 35–50% identity with mosquito SLC1 family members. The SLC1 EAAT-like transporters in C. quinquefasciatus (CuqEAAT) and A. aegypti (AeaEAAT) appear to be orthologs sharing 88% identity. Similarly, the DCT-like orthologs in these species share 78% identity (Table S1). The intra-species EAAT-DCT sequence identity in these two mosquitos is 63–64%.

The electrophysiological and radiolabel uptake data reported in this study on the C. quinquefasciatus gene product establish it as a dicarboxylate transporter rather than an excitatory amino acid transporter. Such a transport activity has not been previously reported in a eukaryotic SLC1 transporter. The selectivity change is associated with discrete changes in TMD8 residues D444 and T448 (EAAT3 numbering). Since the corresponding amino acids appear to be conserved in all other eukaryotic SLC1 genes with the exception of the ortholog in the related mosquito A. aegypti, the mosquito dicarboxylate transporters likely arose in a recent common ancestor of these two species. It is unlikely that these genes are orthologs of the prokaryotic DctA subfamily genes because the latter are absent in other eukaryotic species and the homology of the mosquito DCT transporters to the prokaryotic DctA subfamily (∼25% identity) is lower than with other eukaryotic SLC1 EAAT-like genes (∼30% identity). It thus seems likely that the mutations represent an example of evolution restoring a dicarboxylic acid transport function that was lost with the disappearance of the DctA ortholog from eukaryotic lineages.

Dicarboxylate transport in other eukaryotic organisms is mediated by a subset of the transporters encoded by the structurally unrelated SLC13 gene family [33]. SLC13 orthologs are also found in Culex and Aedes, but their functions have not been reported to date. Interestingly, loss-of-function mutations in an SLC13 dicarboxylate transporter from Drosophila (INDY; I’m not dead yet) are associated with insect lifespan extension [34], [35], raising questions about metabolomic consequences of a gain of function in dicarboxylate uptake through another route. Gaining a better understanding of the molecular strategies for nutrient transport and metabolism that operate in disease-transmitting mosquitos is a key requirement for identifying new targets for their control.

**Figure 5:**
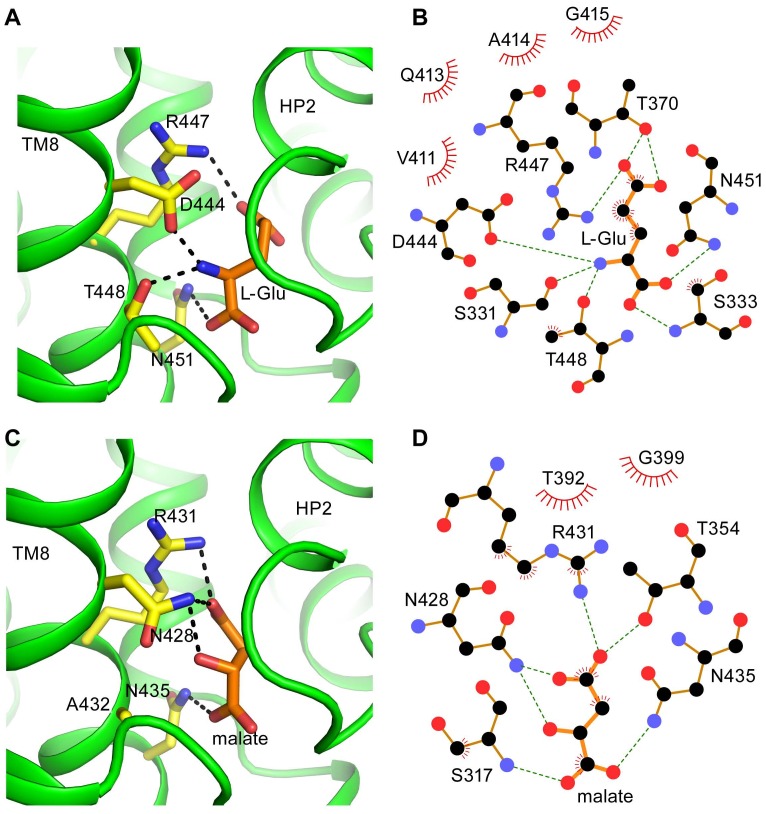
Substrate binding domain models. Binding domain models for human EAAT3 (A,B) and CuqDCT (C,D) (see methods). The models highlight key residues in TMD8 (yellow sticks) and substrate (orange sticks). Conserved side chains in both transporters (R447/R431 and N451/N435) proposed to coordinate distal and proximal carboxylate groups of substrates by electrostatic interaction and H-bonding, respectively. Differences in the key residues (T448/A432 and D444/N428) may contribute to selectivity changes by altering interactions with substrate. Schematic diagrams generated by LIGPLOT indicate possible hydrogen bond interactions between L-glu and EAAT3 (B), and malate and CuqDCT (D).

One of the key structural differences between the mosquito SLC1 DCT orthologs and other eukaryotic SLC1 subfamilies is the presence of an asparagine residue in CuqDCT TMD8 (N428) that is stringently conserved as aspartate in other eukaryotes. Functional data from this study together with previous structural [5] and functional [10] data suggest that in the EAATs, this aspartate residue may interact with the α-amino group of the substrate and contribute to the transporters’ selectivity for α-amino acids over homologous dicarboxylic acids (Fig. 5A, B). Consistent with such a role, this residue is also conserved in the ASCT subfamilies that mediate uptake of neutral α-amino acids. The data from the present study also suggest a role for the residue approximately one helix turn below D444 in TMD8 (T448 in EAAT3) in controlling amino acid/dicarboxylate transport selectivity. In accord with previous results from Teichman and Kanner [10], we found that the single mutation D444S did not confer ability to mediate dicarboxylate transport in EAAT3, despite allowing low-affinity dicarboxylate binding. Moreover, the single mutation T448A neither conferred carboxylate binding nor abolished glutamate transport [10]. The present data show that dicarboxylate-selective transport in EAAT3 required mutation of both T448 and D444. Two mutations were thus likely required for the ancestral mosquito glutamate transporter to evolve dicarboxylate selectivity. The data suggest that these mutations could have proceeded without a loss of transport activity if the sequence of mutations was T->A followed by D->N, but not the converse.

A constrained EAAT3 model based on the Glt_Ph_ structure and these structure function data and highlights possible interactions between four key residues in TMD8 (D444, R447, T448, and N451) and bound substrate (Fig. 5A). Two of these residues are stringently conserved in SLC1 transporters including CuqDCT: R431 (corresponding to R447 in EAAT3) and N435 (corresponding to N451 in EAAT3, Fig. 5C). In addition to structural data [5], evidence from biochemical and electrophysiological studies support the hypothesis that R447 interacts with the distal carboxylate of bound Asp/Glu in the EAATs [12], [36], and this interaction is expected to be maintained in dicarboxylate binding to CuqDCT (Figure 5C,D). Four residues away, close to one helix turn below R447, N451 is positioned to interact with the α-carboxyl group of the bound amino acid in Glt_Ph_ [5]. This is congruous with recently published work showing that mutations in N451 of EAAT3 abolished transport or drastically reduced affinity for L-Glu/L-Asp [37]. The CuqDCT residues corresponding to the identical residues R447 and N451 in EAAT3 may both share analogous functions by stabilizing the two acidic groups of the bound dicarboxylate. In contrast, differences between the CuqDCT residues N428 and A432 and the corresponding EAAT3 residues D444 and T448 appear to be responsible for the change in selectivity exhibited by the novel mosquito dicarboxylate transporter (Figure 5). Hydrogen bonding with N428 may help facilitate dicarboxylate binding in CuqDCT, while the N428D mutation could electrostatically interfere with dicarboxylate binding (Fig. 5C, 5D). In the bacterial DctA family, the serine residue found in the equivalent 428 position could function similarly as a hydrogen bond donor for a bound dicarboxylate ion.

The results of this study also shed light on a potential K^+^ binding site in EAATs. Counter-flux of K^+^ during the transport cycle is a hallmark of EAAT function [28] but the structural determinants involved in the mechanism of countertransport are not well understood. Electrostatic models of EAAT3 suggests that D444 may be involved in coordinating K^+^ during the return transport hemicycle following release of glutamate and Na^+^ [37]. Mutation of the corresponding residue in CuqDCT (N428D) conferred electrophysiological response to K^+^ and the D444S mutation in EAAT3 abolished its response to K^+^. These results provide supporting evidence for an overlapping and mutually exclusive binding site for glutamate with Na^+^ ions or else a K^+^ ion during a transport hemicycle, a simple mechanism that could underlie the thermodynamic coupling of these countertransported ions.

## Supporting Information

Figure S1 **Sequence Alignment.** Multiple sequence alignment for SLC1 transporters from human, insect, bacterial, and archael transporters. CuqDCT, CuqEAAT, AeaDCT, and AeaEAAT represent the EAAT and DCT orthologs from Culex quinquefasciatus and, Aedes aegypti, respectively. AngEAAT1 and AngEAAT2 represent the transporters from Anopheles gambiae. Drosophila EAAT1/2 are from Drosophila melanogaster, Gtl_ph_ from Pyrococcus horikoshii, and DctA is from Bacillus subtillis.
(TIFF)

Figure S2 **Wild-type and mutant EAAT3 dicarboxylic acid uptake.** Uptake of [3H] succinate (10 µM) by oocytes expressing wild-type EAAT3 and mutant EAAT3 D444S/T448A.
(TIF)

Table S1 Sequence identity of SLC1 transporters from various species.
(TIF)
