# Supplementary figures and images for: Novel Dicarboxylate Selectivity in an Insect Glutamate Transporter Homolog
Source: PLoS One. 2013 Aug 7;8(8):e70947. doi: 10.1371/journal.pone.0070947 (PMC3737229; doi:10.1371/journal.pone.0070947)

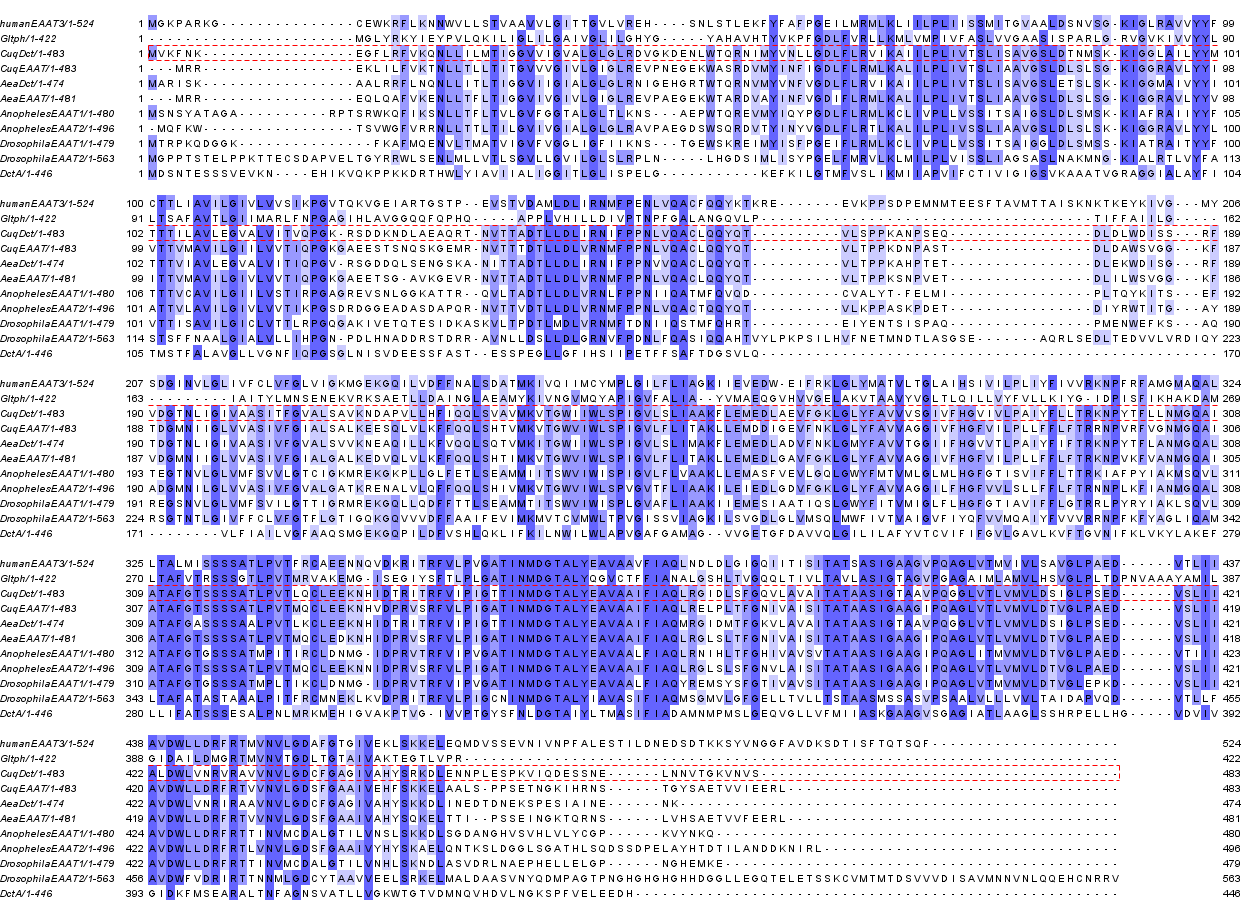

Supplement: Figure S1 — Sequence Alignment. Multiple sequence alignment for SLC1 transporters from human, insect, bacterial, and archael transporters. CuqDCT, CuqEAAT, AeaDCT, and AeaEAAT represent the EAAT and DCT orthologs from Culex quinquefasciatus and, Aedes aegypti, respectively. AngEAAT1 and AngEAAT2 represent the transporters from Anopheles gambiae. Drosophila EAAT1/2 are from Drosophila melanogaster, Gtlph from Pyrococcus horikoshii, and DctA is from Bacillus subtillis. (TIFF) [file pone.0070947.s001.tiff]

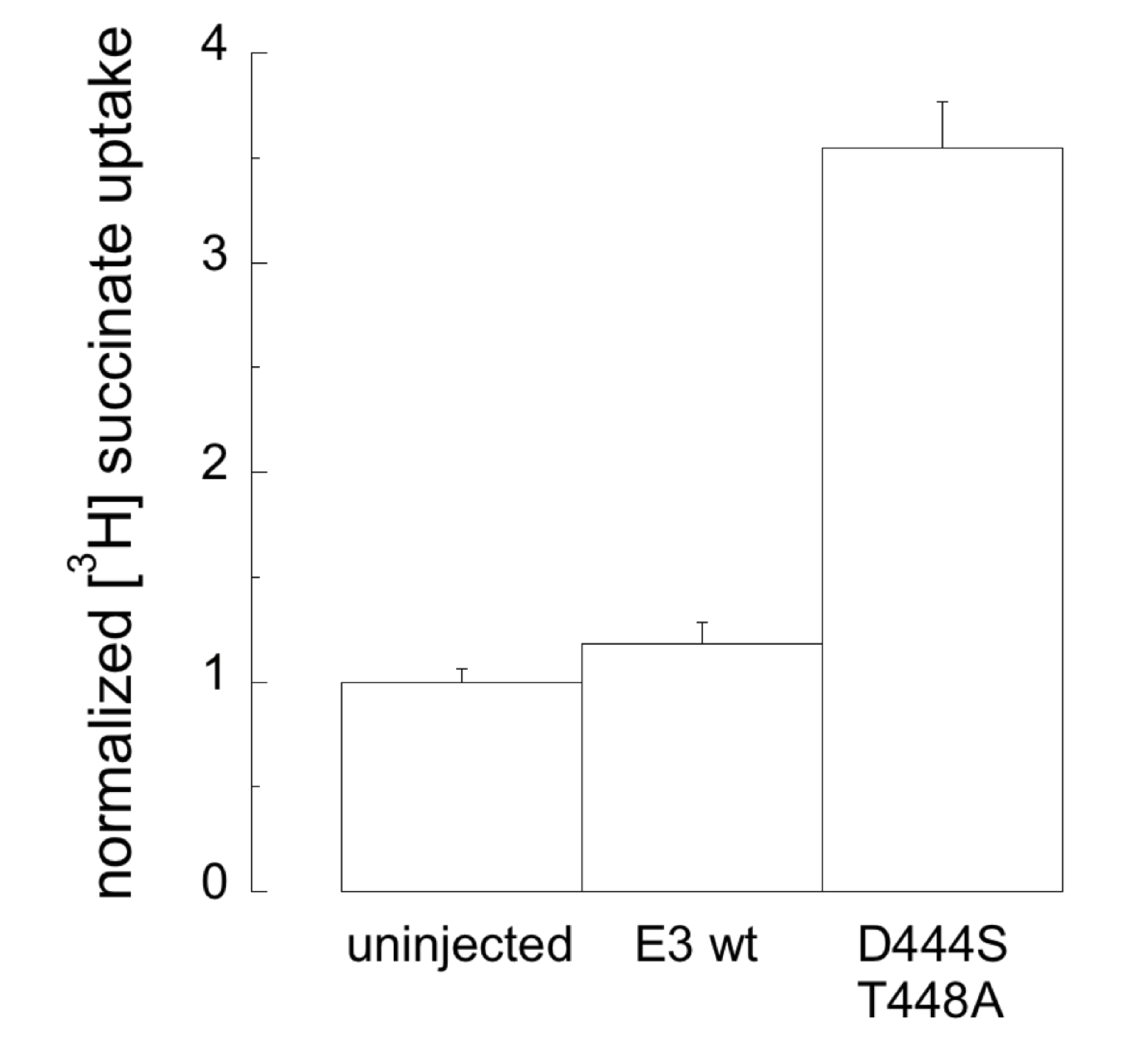

Supplement: Figure S2 — Wild-type and mutant EAAT3 dicarboxylic acid uptake. Uptake of [3H] succinate (10 µM) by oocytes expressing wild-type EAAT3 and mutant EAAT3 D444S/T448A. (TIF) [file pone.0070947.s002.tif]

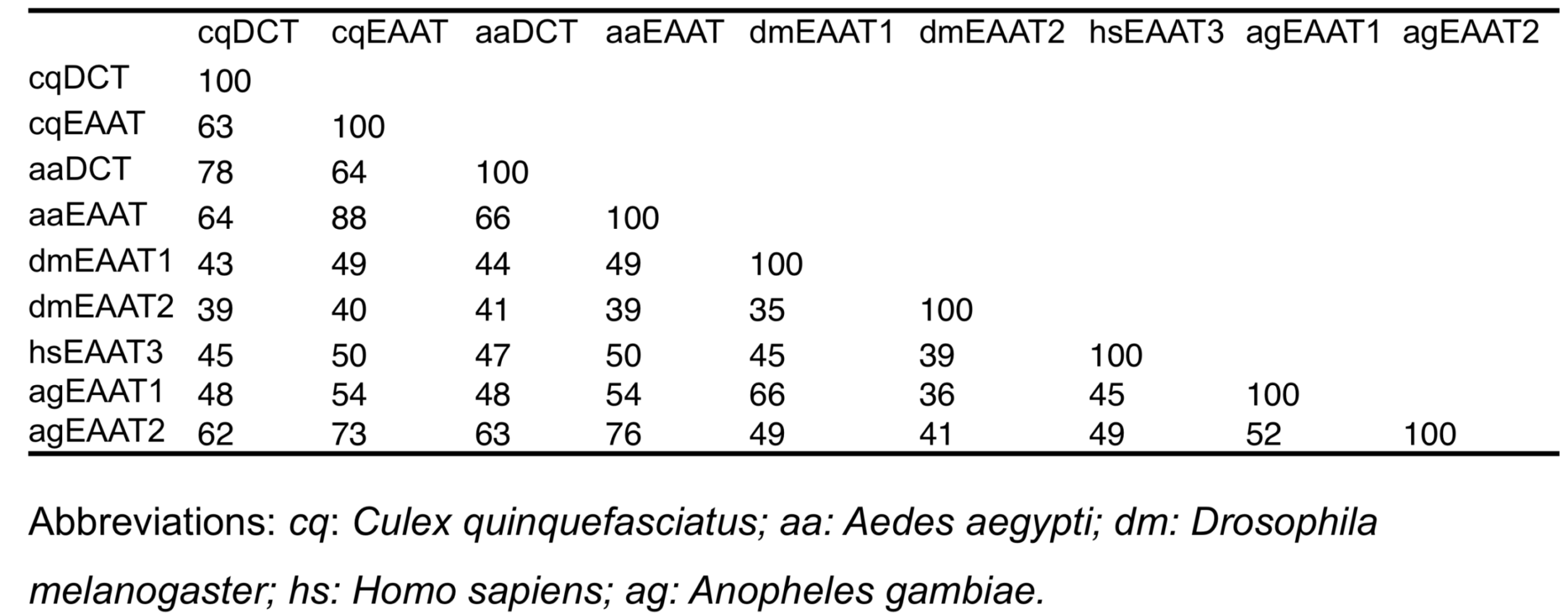

Supplement: Table S1 — Sequence identity of SLC1 transporters from various species. (TIF) [file pone.0070947.s003.tif]
